# Letter from the Editor in Chief

**DOI:** 10.19102/icrm.2019.100401

**Published:** 2019-04-15

**Authors:** Moussa Mansour


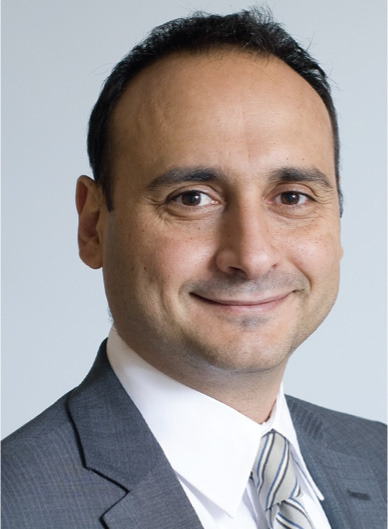


Dear Readers,

The use of ventricular assist devices in patients with congestive heart failure (CHF) has been rapidly increasing. Today, a large number of patients are living with these devices, which are now being used as destination therapy rather than simply a bridge to cardiac transplantation. Importantly, patients with severe left ventricular dysfunction and assist devices often also have ventricular arrhythmias that are challenging to treat.

This issue of *The Journal of Innovations in Cardiac Rhythm Management* contains an excellent article by Drs. Arkles and Marchlinski describing their approach to the treatment of ventricular arrhythmias in these unique individuals. Their manuscript, titled “When Should the Electrophysiologist be Involved in Managing Patients with Ventricular Assist Devices and Ventricular Arrhythmias?,”^1^ provides guidance to the practicing electrophysiologist on how to approach these patients. In it, the authors start by describing the benefit of involving cardiac electrophysiologist specialists at different stages of treatment and then demonstrate the benefits of the early involvement of cardiac electrophysiologists before the mechanical assist device is even implanted. They also explain the mechanisms of different types of ventricular tachycardias in this setting. Most importantly, they provide a practical guide to the treatment of ventricular tachycardia and ventricular fibrillation in this special patient population, including technical details on how to perform catheter ablation and how to navigate different types of challenges that may arise.

I enjoyed reading the abovementioned article because of its clarity and outlined elegant and practical approach to a complex problem. I am confident that you too will find its content educational and relevant to your daily practice.

Sincerely,


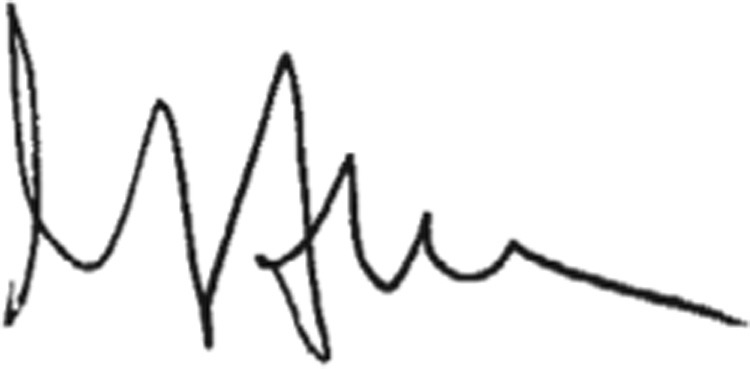


Moussa Mansour, MD, FHRS, FACC

Editor in Chief

The Journal of Innovations in Cardiac Rhythm Management

MMansour@InnovationsInCRM.com

Director, Atrial Fibrillation Program

Jeremy Ruskin and Dan Starks Endowed Chair in Cardiology

Massachusetts General Hospital

Boston, MA 02114

## References

[1] Arkles JS, Marchlinski F (2019). When should the electrophysiologist be involved in managing patients with ventricular assist devices and ventricular arrhythmias?. J Innov Cardiac Rhythm Manage.

